# Advanced lung cancer inflammation index as a new predictor for colon cancer in elderly patients: an NHANES-based study

**DOI:** 10.3389/fnut.2025.1642913

**Published:** 2025-09-04

**Authors:** Hangyan Zhong, Yisheng Chen, Weigen Wu, Suhua Liu, Youlong Fan, Haiqin Liu, Rongqi Lin, Junjie Wan, Meifang He

**Affiliations:** ^1^Laboratory of General Surgery, The First Affiliated Hospital, Sun Yat-Sen University, Guangzhou, China; ^2^Department of Proctology, Shanghang Hospital of Traditional Chinese Medicine, Longyan, Fujian Province, China; ^3^Department of Neurology, Shanghang County Hospital, Longyan, Fujian Province, China; ^4^Center of Hepato-Pancreato-Biliary Surgery, The First Affiliated Hospital, Sun Yat-sen University, Guangzhou, China; ^5^Department of Internal Medicine, Shanghang Hospital of Traditional Chinese Medicine, Longyan, Fujian Province, China; ^6^Department of Internal Medicine, Shanghai Seventh People’s Hospital, Shanghai, China; ^7^Department of Pharmacy, Shanghang County Hospital, Longyan, Fujian Province, China; ^8^National Cancer Center/National Clinical Research Center for Cancer/Cancer Hospital and Shenzhen Hospital, Chinese Academy of Medical Sciences and Peking Union Medical College, Shenzhen, China

**Keywords:** inflammation, nutrition, inflammation index of advanced cancer, colon cancer, NHANES

## Abstract

**Background:**

Nutritional and inflammatory status have both been implicated in colon cancer risk. The advanced lung cancer inflammation index (ALI) is a composite prognostic index that incorporates body mass index (BMI), serum albumin, and neutrophil-to-lymphocyte ratio (NLR), reflecting both nutritional and systemic inflammatory states. However, its role in predicting colon cancer prevalence in elderly individuals remains unclear.

**Methods:**

We used the ALI as a composite marker reflecting both inflammation status and nutritional health. The ALI is calculated as BMI × serum albumin/NLR, where higher values indicate better nutritional status and lower systemic inflammation. To evaluate the association between ALI and colon cancer prevalence, we conducted multivariate logistic regression, applied an Extreme Gradient Boosting (XGBoost) machine learning model, and performed subgroup analyses. Additionally, a smoothed two-piece logistic regression model was used to identify the ALI threshold predictive of colon cancer.

**Results:**

The study included 10,137 elderly participants, with a colon cancer prevalence of 2.45%. The ALI was significantly lower in the colon cancer group compared to those without (*p* < 0.001). Multivariable logistic regression revealed a significant inverse association between ALI and colon cancer (*p* < 0.05), with individuals in the highest ALI tertile experiencing a 67% lower risk compared to those in the lowest tertile (*p* for trend = 0.008). Generalized additive models showed a linear relationship, identifying an inflection point at 4.73 and a predictive threshold of 113.3. Sensitivity analyses confirmed the robustness of these findings, particularly among individuals aged over 70 years, females, unmarried individuals, alcohol consumers, and those with a BMI below 30. In the XGBoost model, ALI demonstrated the highest predictive value for colon cancer (AUC = 0.717), outperforming traditional demographic and clinical variables such as age and BMI. Furthermore, ALI showed a positive association with dietary health status (*p* < 0.05) but was not significantly related to bowel habits.

**Conclusion:**

This study demonstrated that ALI, a nutritional-inflammation prognostic index, is significantly and inversely associated with the risk of colon cancer in older adults. Thus, ALI may serve as a promising, non-invasive biomarker for risk stratification, particularly among high-risk subgroups such as unmarried females, alcohol consumers, and those with lower BMI. Its strong predictive value, confirmed by machine learning, supports its potential role in personalized prevention. Further studies are required to explore underlying mechanisms, including dietary and microbial factors.

## 1 Introduction

Colon cancer is one of the most common malignancies worldwide, with both incidence and mortality rates increasing steadily each year (1). In the United States, this upward trend is particularly evident among older adults, with both the incidence and mortality of colon cancer rising significantly (2). The influence of colon cancer is influenced by multiple factors, including genetics, diet, intestinal inflammation, and intestinal microbiota. Studies have also shown associations between colon cancer and factors such as obesity and marital status (3). This emphasizes the need to distinguish between modifiable (e.g., obesity, physical inactivity, dietary habits) and non-modifiable risk factors (e.g., age, sex, genetics) in developing effective prevention strategies. Inflammation is a key factor in tumor progression (4), as persistent inflammatory responses not only promote tumor cell proliferation but also impair the immune system’s ability to detect and eliminate malignant cells (5). Growing evidence suggests that inflammatory markers may serve as effective predictors of colon cancer risk (6). For example, the systemic immune-inflammation index (SII) and neutrophil-to-lymphocyte ratio (NLR) have been identified as important factors of both the incidence and prognosis of colon cancer (7). In addition, poor nutritional status can contribute to chronic intestinal inflammation, which may further promote cancer cell proliferation (8). Therefore, assessing the combined influence of nutrition and inflammation could offer valuable insight for developing clinical strategies aimed at reducing the risk of colon cancer. The advanced lung cancer inflammation index (ALI) differs from previously established markers by incorporating not only the NLR and albumin (Alb), but also body mass index (BMI) (9), thereby providing a more comprehensive representation of both inflammation and nutritional status in patients with advanced lung cancer (10). This has demonstrated superior prognostic performance compared to other inflammation- and nutrition-based indices (11).

Compared to traditional nutritional risk indices such as the Buzby index or the Geriatric Nutritional Risk Index (GNRI), ALI offers several advantages. First, ALI integrates both inflammatory (NLR) and nutritional (Alb and BMI) components, providing a more comprehensive assessment of patient status. Second, it is simple to calculate using routinely available clinical data. Third, ALI has been validated in various malignancies and chronic diseases as a robust predictor of survival outcomes, making it a clinically practical and prognostically meaningful tool. Emerging evidence also suggests that ALI may serve as a prognostic indicator in breast cancer (12). Previous studies indicated that lower ALI values are associated with poorer survival outcomes in patients with colorectal and gastric cancers, supporting its role as a nutritional prognostic index in these malignancies (13). Importantly, higher ALI values indicate better nutritional status and lower inflammation, which may be protective against cancer risk and progression. Besides oncology, ALI has been widely applied to assess various inflammation- and nutrition-related diseases, including Crohn’s disease, coronary artery disease, and heart failure (14). However, despite its growing clinical relevance, the relationship between ALI and colon cancer remains insufficiently explored. To address this gap, the present study aimed to investigate the association between ALI and colon cancer in elderly individuals in the United States, using data from the 1999–2020 National Health and Nutrition Examination Survey (NHANES).

## 2 Methods

### 2.1 Study population

This study used data from the NHANES database, which employs interviews and physical examinations to evaluate the health and nutritional status of adults and children in the U.S. Since 1999, it has been conducted biennially. The survey protocol was approved by the Research Ethics Committee of the National Center for Health Statistics (NCHS) and the Centers for Disease Control and Prevention (CDC). A written informed consent was obtained from all participants or their legal representatives.

For this analysis, we extracted NHANES data from 1999 to 2020 from individuals aged 60 to 85 years. It should be noted that 85 years was not a strict upper inclusion limit; instead, it reflects the maximum age recorded in publicly available NHANES data due to confidentiality constraints. Participants were excluded if they lacked information on cancer diagnosis, nutritional and inflammatory status, education level, marital status, family income, BMI, ethnicity, smoking, alcohol consumption, or other relevant variables, such as C-reactive protein (CRP), AlB, NLR, and other sociodemographic indicators. Additionally, only datasets with a missing data rate below 20% were included. After applying these criteria, a total of 10,137 eligible participants aged 60 years and older were included in the final analysis (Supplementary Figure S1).

### 2.2 Evaluation of the ALI

ALI was calculated using the formula ALI = BMI × Alb/NLR, where NLR = absolute neutrophil count/absolute lymphocyte count (15). Based on the calculated ALI values, participants were categorized into three groups: low (<61.67), moderate (61.67–84.90), and high (>84.90). These cut-off points correspond to the 33rd and 66th percentiles of the ALI distribution within the study population. Although geriatric-specific nutritional indices like the GNRI have been widely used in older populations, we selected the ALI in this study due to its validated utility across various age groups and its ability to comprehensively reflect both systemic inflammation and nutritional status. In addition, the ALI is a novel and convenient single-index marker that has not yet been reported in the context of colorectal cancer. Given the pivotal role of chronic inflammation in CRC development, the ALI may provide additional clinical insight beyond age-specific nutritional risk scores.

### 2.3 Assessment of colon cancer

Data were obtained from the Medical Conditions Questionnaire, in which participants were first asked, “Have you ever been told by a doctor or other health professional that you have cancer or any malignancy?” Those who answered “yes” were then asked to specify the type of cancer. Patients who reported only colon cancer (including both primary and isolated tumors) were classified as having colon cancer. In contrast, individuals who reported no history of cancer, a history of other malignancies, or colon cancer combined with other cancer types were categorized as not having colon cancer (16).

### 2.4 Covariate selection

Based on a review of relevant literature and clinical knowledge, we identified a set of covariates associated with colon cancer to include in our study (Supplementary Figure S2). These covariates included sex (male or female), age (continuous), race/ethnicity (Mexican American, other Hispanic, non-Hispanic White, non-Hispanic Black, and other races), smoking status (yes/no), alcohol consumption (yes/no), education level (less than high school, high school or equivalent, and more than high school), BMI (<25 kg/m^2^, 25–30 kg/m^2^, and >30 kg/m^2^), marital status (married/living with partner, widowed/divorced/separated, and never married), and poverty-to-income ratio (PIR) (<1.30, 1.30–3.50, and >3.50). Dietary health status was assessed using the NHANES variable DBQ700, which asked participants to self-assess the overall healthfulness of their diet. Responses were categorized as follows: “Good” (responses 1 = Excellent, 2 = Very good, 3 = Good), “Fair” (response 4), “Poor” (response 5). Participants who responded “Refused,” “Do not know,” or had missing values were excluded. Stool type and defecation frequency were derived from the NHANES variable BHQ060. Chronic constipation was defined as responses 1–2, chronic diarrhea as responses 6–7, and normal stool patterns as responses 3–5. Participants with missing, unknown, or refused answers were excluded. We assessed the normality of continuous variables using the Shapiro–Wilk test and visual inspection of histograms.

### 2.5 Statistical analysis

The analyzed data were weighted following the guidelines provided by NCHS. Due to the marked imbalance between the number of colon cancer cases and controls, we applied NHANES-recommended sampling weights in all analyses to reduce potential bias. In addition, sensitivity analyses including subgroup analyses and propensity score matching (PSM) were conducted to further address group imbalance and assess the robustness of our findings. Participants were divided into two groups based on the presence or absence of colon cancer. Continuous variables are presented as medians with interquartile ranges (IQRs), and categorical variables are expressed as counts and percentages. Nonparametric tests were used to compare continuous variables, while chi-square tests were applied to compare categorical variables. The ALI values were log-transformed using base 10 logarithm (log₁₀) to normalize their distribution and analyzed both as a continuous variable and as tertiles in regression models. Logistic regression models were employed to examine the association between ALI and colon cancer, and a linear trend test was conducted to evaluate the consistency of this relationship across tertiles. All multivariable models were adjusted for key covariates based on clinical relevance and data availability. These included demographic variables (age, sex, race/ethnicity, marital status, education level, and family income-to-poverty ratio [PIR]); lifestyle factors (smoking status and alcohol consumption); anthropometric indicators (BMI); and inflammatory/nutritional markers (serum albumin and C-reactive protein [CRP]). To further explore the relative contribution of various factors to colon cancer incidence, we applied an Extreme Gradient Boosting (XGBoost) machine learning algorithm. The model was trained with a learning rate of 0.3, a maximum tree depth of 8, and 500 trees to optimize performance while minimizing overfitting. Hyperparameter tuning was performed via 10-fold cross-validation (17, 18). The predictive performance of each variable was evaluated using the area under the receiver operating characteristic curve (AUC), mean squared error (MSE), and root mean squared error (RMSE). To interpret the model’s internal logic and assess the contribution of each predictor, we used Shapley Additive Explanations (SHAP) values. These values provide an interpretable summary of variable importance by quantifying each feature’s impact on model output across all permutations. To evaluate the potential nonlinear relationship between ALI and colon cancer, a restricted cubic spline (RCS) curve was generated. Based on this curve, the inflection point was identified using a recursive algorithm, and a two-piece linear regression model was constructed accordingly. Additionally, the relationship between ALI and intestinal health status was explored to gain further insight into the potential mechanisms underlying the association between ALI and colon cancer.

To confirm the reliability of our findings, we conducted a series of sensitivity analyses. First, individuals with other malignant tumors were excluded, and a preliminary analysis was performed to assess potential bias introduced by these participants. Second, subgroup analyses were conducted based on key covariates and their interactions with ALI, with adjustments made for potential confounders. Third, PSM at a 1:1 ratio was performed to balance covariate distributions between the colon cancer group and the control group. Matching variables included sex, age, race/ethnicity, educational level, marital status, family income, and BMI. Following PSM, the matched study population was reanalyzed to verify the robustness of the observed associations. Furthermore, to assess the discriminatory ability of ALI in identifying individuals with colon cancer, we constructed a receiver operating characteristic (ROC) curve and calculated the area under the curve (AUC). This analysis served to validate the predictive performance of ALI in our model beyond its statistical significance in regression. All statistical analyses were performed using R software. A *p*-value < 0.05 was considered statistically significant.

## 3 Results

### 3.1 Baseline characteristics

A total of 10,137 participants were included in this study, among whom 248 were diagnosed with colon cancer, accounting for 2.45%, as shown in Table 1. Compared with the control group, participants with colon cancer were more likely to be older, of non-Hispanic White ethnicity, married or living with a partner, overweight, and to have a lower level of Alb, absolute lymphocyte count, absolute monocyte count, neutrophil/lymphocyte ratio, and low platelet count (*p* < 0.05). The ALI value was significantly lower in the colon cancer group [median (IQR): 55.20 (39.28, 74.16)] compared to the control group [median (IQR): 71.88 (58.18, 94.03)] (*p* < 0.001), suggesting that a lower ALI is associated with poor nutrition and higher systemic inflammation.

**Table 1:** Baseline characteristics of participants by colon cancer status.

Variables	Total (*n* = 10,137)	Control (*n* = 9,889)	Colon cancer (*n* = 248)	*p*-value
Gender, *n* (%)				0.046
Male	4,804 (47.39)	4,671 (47.23)	133 (53.63)	
Female	5,333 (52.61)	5,218 (52.77)	115 (46.37)	
Age M (Q_1_, Q_3_)	68.00 (63.00, 75.00)	68.00 (63.00, 75.00)	76.00 (70.00, 80.00)	<0.001
60–70	5,990 (59.09)	5,920 (59.86)	70 (28.23)	
>70	4,147 (40.91)	3,969 (40.14)	178 (71.77)	
Race, *n* (%)				<0.001
Mexican American	1,505 (14.85)	1,489 (15.06)	16 (6.45)	
Other Hispanic	834 (8.23)	818 (8.27)	16 (6.45)	
Non-Hispanic White	4,924 (48.57)	4,756 (48.09)	168 (67.74)	
Non-Hispanic Black	2,240 (22.10)	2,199 (22.24)	41 (16.53)	
Other race	634 (6.25)	627 (6.34)	7 (2.82)	
Smoke, *n* (%)				0.952
Yes	4,597 (45.35)	4,485 (45.35)	112 (45.16)	
No	5,540 (54.65)	5,404 (54.65)	136 (54.84)	
Alcohol drinker, *n* (%)				0.780
Yes	8,394 (82.81)	8,187 (82.79)	207 (83.47)	
No	1743 (17.19)	1702 (17.21)	41 (16.53)	
Education, *n* (%)				0.752
Less than high school	3,334 (32.89)	3,247 (32.83)	87 (35.08)	
High school or equivalent	2,443 (24.10)	2,386 (24.13)	57 (22.98)	
College or above	4,360 (43.01)	4,256 (43.04)	104 (41.94)	
Marital status, *n* (%)				0.048
Married/living with partner	5,983 (59.02)	5,855 (59.21)	128 (51.61)	
Widowed/divorced/separated	3,787 (37.36)	3,676 (37.17)	111 (44.76)	
Never married	367 (3.62)	358 (3.62)	9 (3.63)	
PIR, *n* (%)				0.628
<1.30	2,952 (29.12)	2,881 (29.13)	71 (28.63)	
1.30–3.50	4,423 (43.63)	4,308 (43.56)	115 (46.37)	
>3.50	2,762 (27.25)	2,700 (27.30)	62 (25.00)	
BMI M (Q_1_, Q_3_)	29.20 (25.98, 33.30)	29.20 (26.00, 33.33)	27.98 (25.27, 32.56)	0.008
Normal weight (<25 kg/m^2^)	1,858 (18.33)	1,801 (18.21)	57 (22.98)	
Overweight (25–30 kg/m^2^)	3,816 (37.64)	3,718 (37.60)	98 (39.52)	
Obese (>30 kg/m^2^)	4,463 (44.03)	4,370 (44.19)	93 (37.50)	
CRP M (Q_1_, Q_3_)	0.39 (0.21, 1.02)	0.39 (0.21, 1.02)	0.39 (0.19, 0.93)	0.427
Alb M (Q_1_, Q_3_)	4.20 (4.00, 4.40)	4.20 (4.00, 4.40)	4.10 (3.90, 4.30)	<0.001
Lymph M (Q_1_, Q_3_)	2.10 (1.80, 2.60)	2.20 (1.80, 2.60)	1.80 (1.40, 2.20)	<0.001
Mono M (Q_1_, Q_3_)	0.50 (0.40, 0.70)	0.50 (0.40, 0.70)	0.60 (0.50, 0.70)	0.047
Neut M (Q_1_, Q_3_)	3.60 (2.80, 4.50)	3.60 (2.80, 4.40)	3.90 (3.00, 5.00)	<0.001
PLT M (Q_1_, Q_3_)	236.00 (199.00, 279.00)	236.00 (199.00, 279.00)	229.00 (188.00, 271.00)	0.027
ALI M (Q_1_, Q_3_)	71.42 (57.86, 93.57)	71.88 (58.18, 94.03)	55.20 (39.28, 74.16)	<0.001

Continuous variables were analyzed using the Wilcoxon test and expressed as median (IQR), whereas categorical variables were analyzed using the Chi-square test and presented as *n* (%). IQR, interquartile range; PIR, poverty-income ratio; BMI, body mass index; CRP, C-reactive protein; Alb, serum albumin (biochemical test); Lymph, absolute lymphocyte count (CBC); Mono, absolute monocyte count (CBC); Neut, absolute neutrophil count (CBC); PLT, absolute platelet count (CBC); ALI, advanced lung cancer inflammation index. M: Median; Q1: First quartile; Q3: Third Quartile.

### 3.2 Correlation between ALI and colon cancer incidence

After log transformation, ALI was analyzed both as a continuous and a categorical variable. The association between log ALI and the incidence of colon cancer was analyzed using logistic regression, as presented in Table 2. A significant inverse relationship was observed between ALI and colon cancer across all models, with odds ratios in Model 1 (0.03 [0.02–0.05]), Model 2 (0.05 [0.03–0.07]), and Model 3 (0.05 [0.03–0.07]). Sensitivity analysis using tertile-based categorization of log-transformed ALI further supported this finding. Participants were categorized into tertiles based on the empirical distribution of log ALI values: T1 (<2.95), T2 (2.95–3.45), and T3 (>3.45). In both Models 2 and 3, individuals in T3 had approximately a 67% lower risk of colon cancer compared to those in T1 (Model 3: OR = 0.33, 95% CI: 0.17–0.65; P-value for trend = 0.008).

**Table 2:** Association of advanced log ALI among the US participants aged 60 to 85 years, NHANES, 1999 to 2020.

log ALI	Control (*n* = 9,889)	Colon cancer (*n* = 248)	Model 1	*p*-value	Model 2	*p*-value	Model 3	*p*-value
Per ln-unit increase			0.03 (0.02 ~ 0.05)	<0.001	0.05 (0.03 ~ 0.07)	<0.001	0.05 (0.03 ~ 0.07)	<0.001
T1 (<2.95)	3,197 (32.33)	146 (58.87)	1.00 (Reference)		1.00 (Reference)		1.00 (Reference)	
T2 (2.95–45)	3,386 (34.24)	62 (25.00)	0.40 (0.30 ~ 0.54)	<0.001	0.45 (0.33 ~ 0.62)	<0.001	0.45 (0.33 ~ 0.62)	<0.001
T3 (>3.45)	3,306 (33.43)	40 (16. 13)	0.26 (0.19 ~ 0.38)	<0.001	0.33 (0.23 ~ 0.47)	<0.001	0.33 (0.23 ~ 0.47)	<0.001
*p* for trend				<0.001		<0.001		<0.001

Model 1: Crude. Model 2: Adjust: gender, race, education, marital status, PIR, smoke, alcohol drinker. Model 3: Adjust: gender, age, race, education, marital status, PIR, smoke, alcohol drinker. Log-transformation refers to log base 10 (log_10_). OR, odds ratio; CI, confidence interval.

### 3.3 Exploration of nonlinear relationships

Using generalized additive models (GAM) and smoothed curve fitting, we examined the relationship between ALI and colon cancer and found a linear association after adjusting for all variables (nonlinear *p*-value < 0.001; log-likelihood ratio test *p* < 0.001) (Supplementary Figure S3). A comparison between the linear regression model and the two-stage linear regression model (Supplementary Table S1) indicated that the two-stage regression model provided a better fit for the data. Using the recursive method and two-stage regression model, the inflection point was identified as 4.73, and the corresponding ALI threshold was calculated as 113.3, which may represent a potential cut-off value for reducing the risk of colon cancer.

### 3.4 Sensitivity analysis

**Figure 1:**
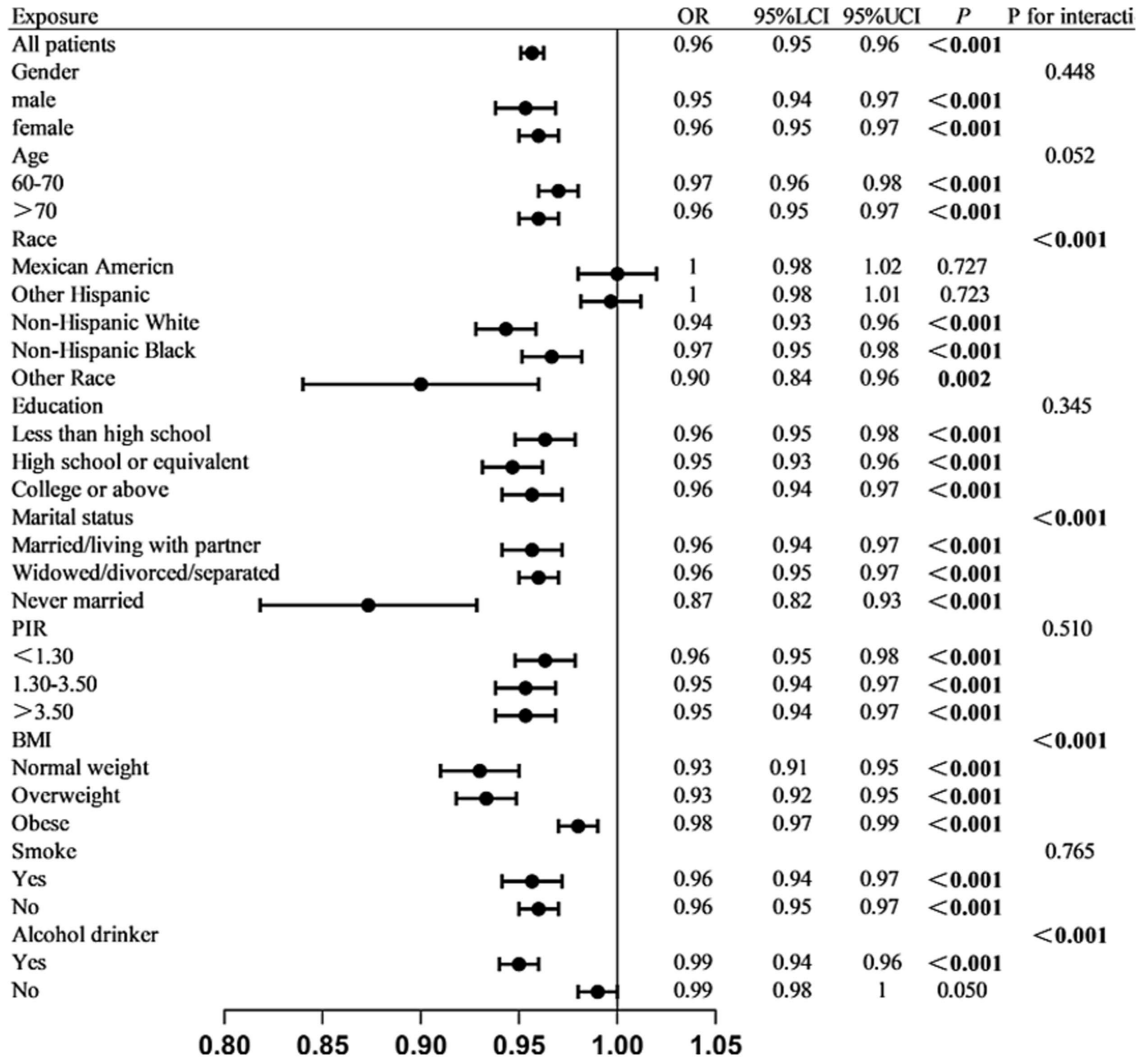
Stratified association between log₁₀-transformed ALI and colon cancer across subgroups. Multivariable logistic regression analyses were conducted in various subgroups to assess the association between ALI and colon cancer. Estimates were adjusted for all covariates listed in the Methods section unless otherwise specified. Interaction *p*-values are provided for subgroup comparisons.

Multiple sensitivity analyses were performed to evaluate the reliability of the findings. First, excluding participants with other malignant tumors from the control group yielded results consistent with the primary analysis (Supplementary Table S2). Second, subgroup analyses demonstrated that higher ALI levels were significantly associated with a reduced risk of colon cancer across various subpopulations, supporting a potential protective effect. The protective effect of ALI was more pronounced among individuals aged over 70 years, females, unmarried individuals, those who consumed alcohol, and those with a BMI <30. The presence of potential interactions among several subgroups indicates that the protective effect of ALI on colon cancer may be modulated by individual metabolic status, lifestyle factors, and socioeconomic conditions. (Figure 1). Subgroup analyses were conducted to examine whether the association between ALI and colon cancer varied across different strata (e.g., age, sex, BMI). Figure 1 presents the odds ratios and 95% confidence intervals of ALI for colon cancer risk across these subgroups. These comparisons are exploratory and should not be interpreted as indicating causal relationships between ALI and the stratifying variables. After performing PSM, 468 participants were retained in this analysis, with 234 individuals in both the colon cancer and control groups. Differences in variables between the groups were noted before and after PSM (Supplementary Figure S4). Regardless of model adjustment, ALI remained significantly associated with colon cancer (Supplementary Table S3).

### 3.5 Predictive analysis via XGBoost model

**Figure 2:**
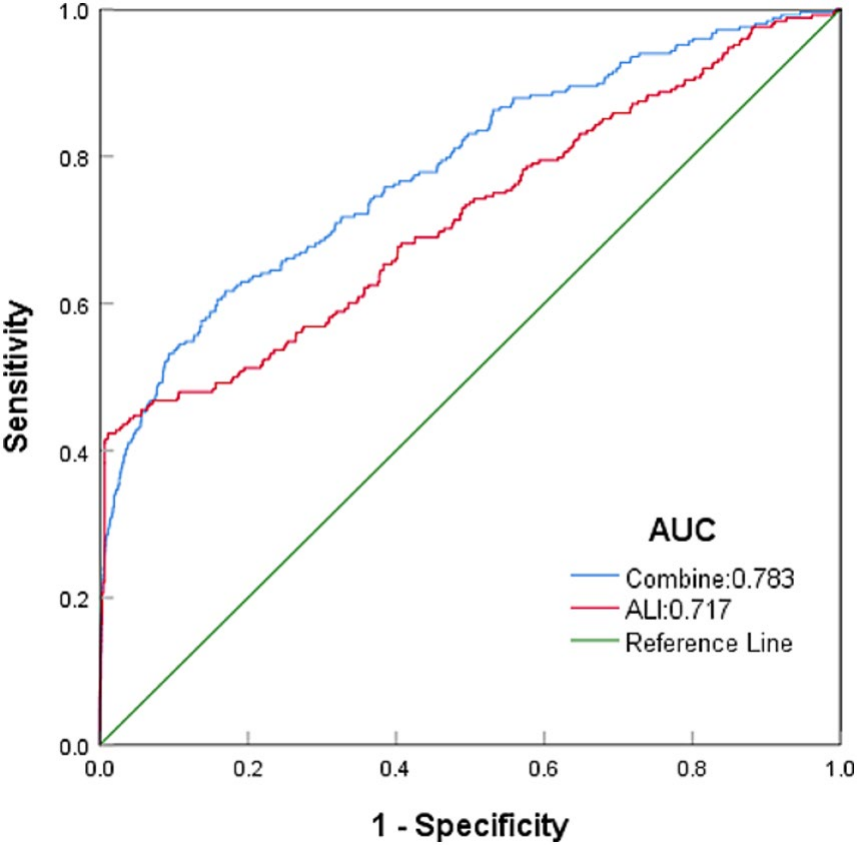
Top predictors of colon cancer identified by the XGBoost model, ranked by AUC. To compare the predictive ability of ALI and other variables, we used the XGBoost machine learning model with 10-fold cross-validation. The AUC values of the top variables are displayed. ALI ranked as the strongest predictor, followed by age and BMI.

**Figure 3:**
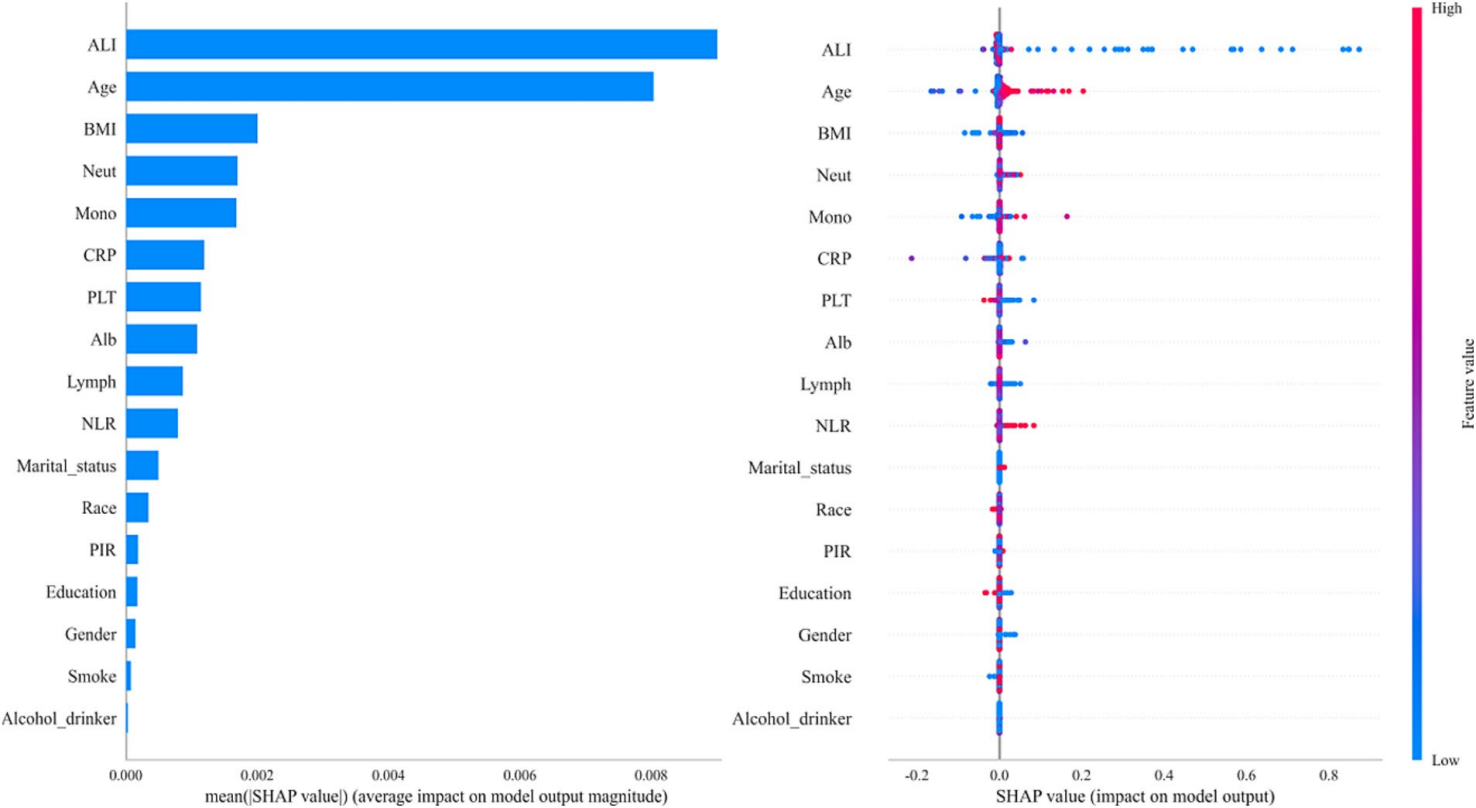
SHAP summary plot demonstrating the contribution of each variable to the XGBoost model. SHAP values were used to interpret the contribution of each feature in the model. The plot illustrates both the magnitude and direction (positive or negative) of each variable’s impact on the predicted colon cancer risk.

Among all the variables analyzed in this study, ALI showed the strongest predictive ability for colon cancer incidence, with an area under the curve (AUC) of 0.717 (Figure 2). Age ranked second with an AUC of 0.712, followed by BMI with an AUC of 0.549. Using the XGBoost machine learning model, the final prediction showed a mean square error (MSE) of 0.08, indicating minimal deviation between predicted and observed values. Additionally, the root mean square error (RMSE) was 0.28, further demonstrating the model’s high predictive accuracy. Figure 3 summarizes the SHAP analysis results of the XGBoost model. ALI emerged as the most influential predictor of colon cancer incidence, followed by age and BMI. The SHAP summary plot provides an interpretable visualization of the contribution of each variable to model predictions. The importance matrix and SHAP dependence plot further confirmed the prominent role of ALI in the model. These findings reinforce the robust predictive value of ALI and highlight its potential utility in early risk stratification for colon cancer.

### 3.6 Investigation of potential links

Furthermore, this study also evaluated the relationship between ALI and dietary health status, stool type, and the frequency of defecation per week after full adjustment for confounders. A significant positive association was observed between dietary health status and ALI in both the total population and the control group (Table 3). In contrast, no significant associations were found between ALI and either stool type or weekly defecation frequency in either group (Supplementary Table S4).

**Table 3:** The association between ALI and dietary health status in the overall population and control group.

Mediating factors	Overall (*n* = 34,452)	*p-*value	Controls (*n* = 34,235)	*p-*value
	β (95% CI)^a^		β (95% CI)^a^	
Good	0.00 (Reference)		0.00 (Reference)	
Fair	−0.01 (−2.55 ~ 2.52)	0.992	0.00 (−2.55 ~ 2.56)	0.998
Poor	9.03 (3.90 ~ 14.15)	<0.001	9. 16 (4.02 ~ 14.31)	<0.001

Dietary health was assessed by self-rated diet quality (DBQ700). Stool type was based on the Bristol Stool Form Scale (BHQ060). Defecation frequency was derived from BHQ100. ^a^Indicates estimates adjusted for covariates listed in the Methods section.

## 4 Discussion

This study demonstrated a strong and consistent inverse linear association between the ALI and the incidence of colon cancer, indicating that higher ALI levels may have a protective effect. Among all variables examined, ALI emerged as the most significant predictor of colon cancer risk, as confirmed by the XGBoost machine learning model. Furthermore, a significant association between ALI and dietary health status was identified, suggesting a potential association between the nutritional-inflammatory balance and colorectal cancer occurrence. These findings suggest that higher ALI levels and healthy dietary patterns are associated with lower colon cancer risk, though causal relationships cannot be established.

Using a smoothed two-stage logistic regression model, the ALI threshold for colon cancer incidence was identified as 113.3. This threshold determination plays a significant clinical role in developing personalized prevention strategies and implementing early interventions for high-risk populations.Subgroup analyses revealed that higher ALI levels were significantly associated with reduced odds of colon cancer in specific populations, suggesting a potential protective effect. This supports the interpretation that higher ALI values reflect better nutritional and lower inflammatory status. This inverse association was particularly evident among individuals aged over 70 years, females, unmarried individuals, those who consumed alcohol, and those with a BMI below 30. These findings may reflect the stronger influence of nutritional and inflammatory imbalance in populations with limited social support (19), or those experiencing alcohol-related metabolic disturbances (20). Although not all subgroup associations reached statistical significance, the consistent negative trend across groups highlights the potential of ALI as a universal prognostic biomarker.

The ALI demonstrates strong predictive power for colon cancer incidence among the variables analyzed in this study. The XGBoost model identified ALI as one of the top three most influential predictors. As an advanced machine learning algorithm, XGBoost can handle large-scale datasets and uncover complex nonlinear relationships among variables. It has been widely validated in many medical studies for its effectiveness in predicting disease risks, particularly in conditions characterized by multi-factor interactions (21, 22).Further exploration of the relationship between ALI and dietary health status, stool type, and weekly defecation frequency revealed a significantly positive correlation between ALI and dietary health status. This association may be attributed to the impact of diet on the intestinal microbiota, which plays a critical role in colon cancer development. For example, diets high in red meat may promote the growth of certain bacteria, such as Klebsiella, Clostridium, and Staphylococcus, which can produce carcinogens and promote intestinal carcinogenesis (23). High-fat diets have been shown to increase the production of secondary bile acids such as deoxycholic acid (DCA) and lithocholic acid (LCA) (24). Similarly, a high-fructose diet has been shown to alter the proliferation and cell growth induced by the intestinal microbiota in pancreatic and colon cancer cell lines (25), whereas high-sugar diets may disrupt the intestinal microbiota balance and facilitate the translocation of bacteria and toxins to enter the bloodstream, thereby promoting cancer development (26). In contrast, diets rich in dietary fiber, low in fat, and abundant in antioxidants and fermented foods can promote the fermentation of intestinal microbiota to produce short-chain fatty acids (SCFAs) such as acetic acid, propionic acid, and butyric acid (27). These SCFAs improve intestinal barrier function and exhibit anti-inflammatory effects, which may lower colon cancer risk (28). Butyric acid, in particular, is considered a potent anti-cancer substance that regulates proliferation, differentiation, and apoptosis of intestinal epithelial cells (29, 30), and also modulates immune responses while reducing inflammation (31, 32). Therefore, considering the close association between gut microbiota dysbiosis and colon cancer, and the role of a healthy diet in preserving microbial homeostasis, our study suggests that the observed association between ALI and colon cancer may be partly explained by its correlation with healthy dietary patterns and potential links with gut microbiota.

Numerous studies have demonstrated a close association between colon cancer and inflammatory responses, with inflammation contributing to tumor development by activating immune pathways and compromising the integrity of the intestinal barrier (33). Patients with inflammatory bowel disease (IBD) exhibit a significantly higher incidence of colon cancer compared to the general population (34). ALI, as a composite marker inflecting both inflammation status and nutritional health, is relevant not only in various types of cancer but also in influencing the onset and prognosis of colon cancer (35). Several studies have highlighted the predictive value of ALI in the development and progression of multiple types of cancer (36). Maintaining good nutritional status plays a critical role in preserving intestinal barrier function, minimizing inflammatory responses, and thereby reducing colon cancer risk (37, 38). The presence of potential interactions among several subgroups suggests that the observed association between ALI and colon cancer may be influenced by lifestyle factors and socioeconomic conditions.

Traditional colorectal cancer (CRC) screening tools, such as carcinoembryonic antigen (CEA) and the fecal immunochemical test (FIT), focus primarily on tumor markers or occult gastrointestinal bleeding. In contrast, the ALI captures a broader physiological profile by incorporating systemic inflammation and nutritional parameters. Although CEA and FIT remain central to early CRC detection, they provide limited information on host-related factors. The ALI, calculated from routine clinical data (BMI, Alb, and NLR), is non-invasive and low-cost, and may serve as a valuable adjunct for risk evaluation in elderly individuals, who often face malnutrition and chronic inflammatory states.

This study has several strengths and limitations. First, XGBoost effectively handles high-dimensional data by automatically selecting the most predictive variables, thereby eliminating the need for manual variable selection used in traditional statistical methods. This approach enhances model accuracy, reduces the risk of overfitting, and improves generalizability to unseen data.By quantifying the contribution of each variable to the model, XGBoost helps researchers to identify key factors associated with colon cancer incidence and reveals complex nonlinear relationships among variables, offering insights for clinical decision-making, prioritizing high-risk factors, and guiding targeted prevention strategies. The combination of XGBoost with clinical practice provides a new perspective for early prevention and intervention of colon cancer and holds significant implications for public health. Second, determining the ALI threshold using an inflection point enhances the ability to identify individuals at elevated risk more accurately. Early preventive measures, such as routine screening and lifestyle modifications including diet and exercise, can be implemented to reduce colon cancer risk. Clinically, using the ALI threshold allows physicians to assess individual colon cancer risk more precisely and, when combined with other risk factors (e.g., age, sex, family history), to develop personalized health management plans. Third, this study suggests that ALI may influence colon cancer risk through its association with dietary health and the gut microbiota. Unhealthy dietary patterns may disrupt the intestinal microbiota and promote carcinogenesis. Exploring the mediating role of the gut microbiota in the ALI–colon cancer association provides novel insights for cancer prevention. These findings not only promote interdisciplinary collaboration but also offer a potential theoretical basis for public health policy development. Future research is needed to clarify the mechanistic role of the gut microbiota in colon cancer pathogenesis, which may enhance strategies for early diagnosis and prevention. Given the rising global burden of colon cancer, exploring how ALI and dietary health affect cancer development through the microbiota has both scientific relevance and practical value for global cancer prevention and health management.

One limitation of this study is the lack of detailed clinical information in the NHANES dataset, such as cancer stage or treatment history. Although we excluded participants with other self-reported malignancies using variable MCQ220 in sensitivity analyses, residual confounding may persist. Additionally, the imbalance in the number of colon cancer cases compared to controls may cause potential estimation bias. To address this, we used weighted logistic regression and conducted multiple sensitivity analyses, including subgroup analyses and propensity score matching, which helped ensure the robustness and reliability of our findings. Another limitation of this study is the lack of direct analysis of dietary patterns and gut microbiota in the study population. Thus, future research should incorporate these factors to explore the underlying mechanisms linking ALI to colon cancer.

## 5 Conclusion

This study, based on a large nationally representative sample, demonstrated a significant inverse association between ALI and the risk of colon cancer in older adults. ALI, calculated from BMI, serum albumin, and NLR, is a nutritional-inflammation prognostic index that reflects both systemic inflammation and nutritional status. Our findings remained robust across multiple analytical models and subgroups. The predictive value of ALI was further validated using an XGBoost machine learning model, in which ALI emerged as the most important feature.

We also found a positive association between ALI and dietary health status, suggesting a link between nutritional-inflammatory balance and colon cancer risk. However, as gut microbiota data were not assessed in this study, any mechanistic speculation involving microbiome pathways remains premature and requires further investigation.

Collectively, these results indicated that ALI may serve as a promising, non-invasive index for stratifying the risk of prevalent colon cancer in elderly adults. Given its accessibility and clinical interpretability, ALI could be integrated into risk-based screening strategies to support personalized cancer prevention. Future prospective studies incorporating detailed dietary data and gut microbiome profiling are needed to clarify the underlying biological mechanisms.

## Data Availability

The original contributions presented in the study are included in the article/Supplementary material, further inquiries can be directed to the corresponding authors.
