# Supplementary figures and images for: Advanced lung cancer inflammation index as a new predictor for colon cancer in elderly patients: an NHANES-based study
Source: Front Nutr. 2025 Sep 4;12:1642913. doi: 10.3389/fnut.2025.1642913 (PMC12445050; doi:10.3389/fnut.2025.1642913)

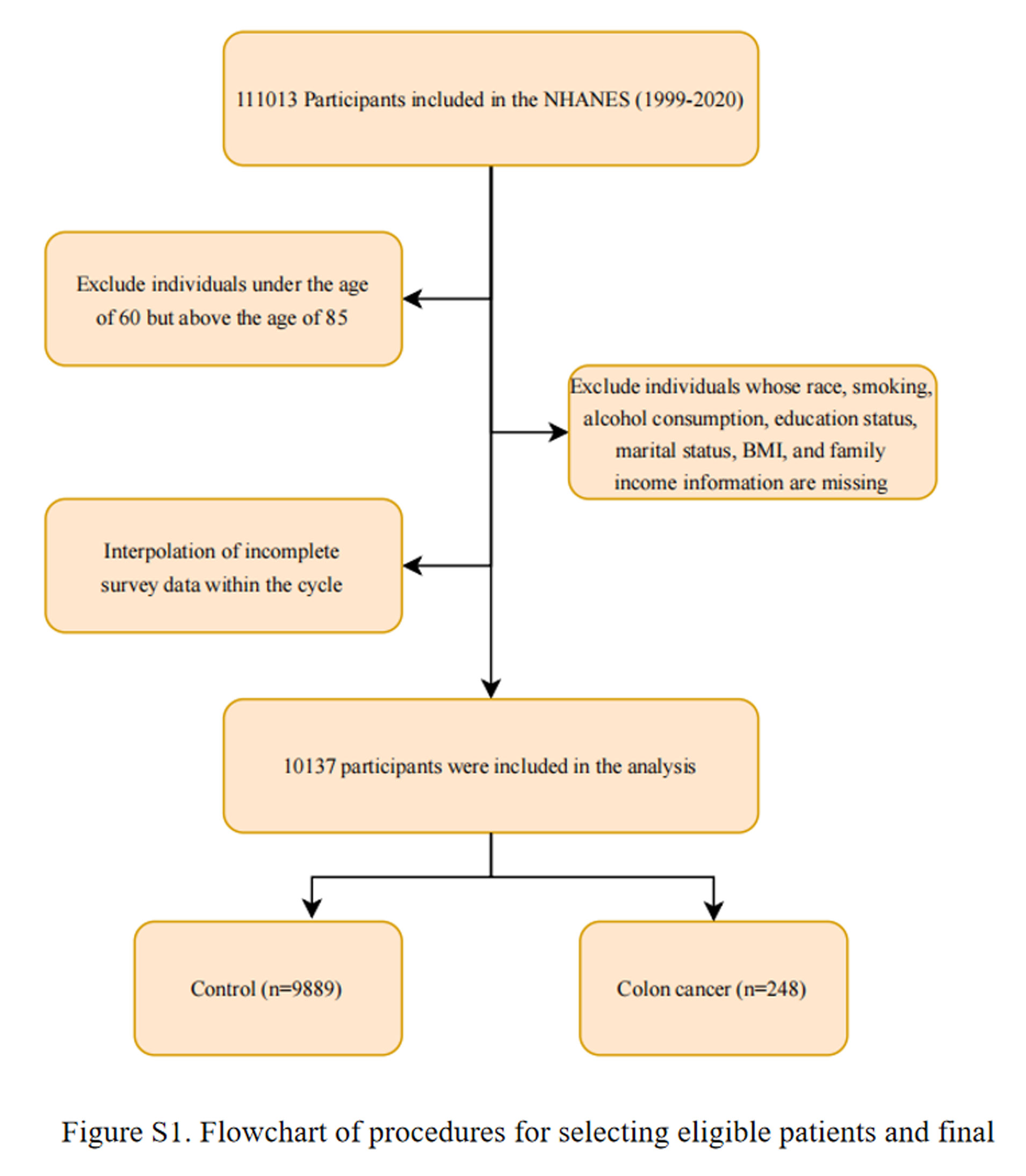

Supplement: Supplementary file 1 [file Image_1.tif]

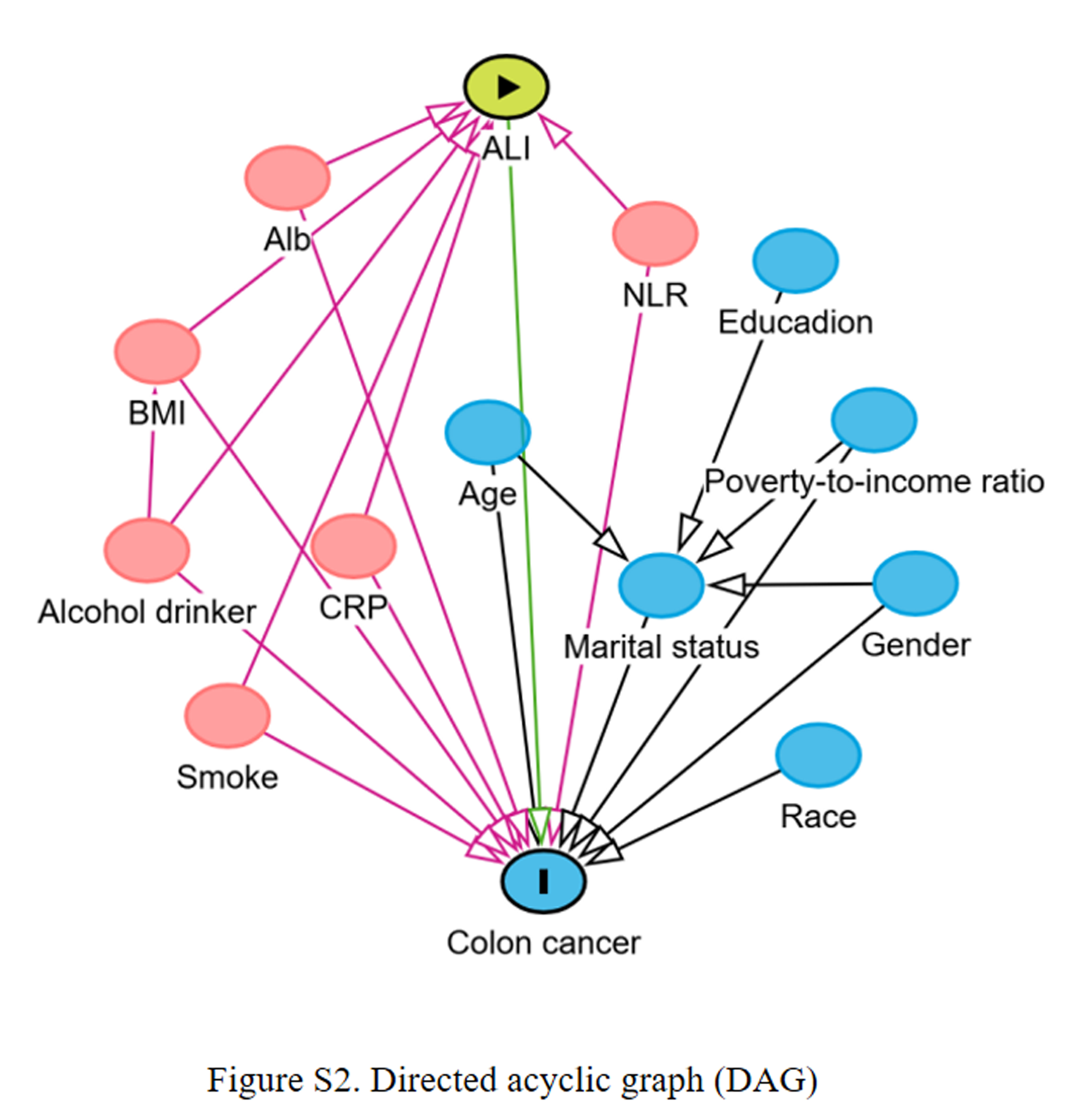

Supplement: Supplementary file 2 [file Image_2.tif]

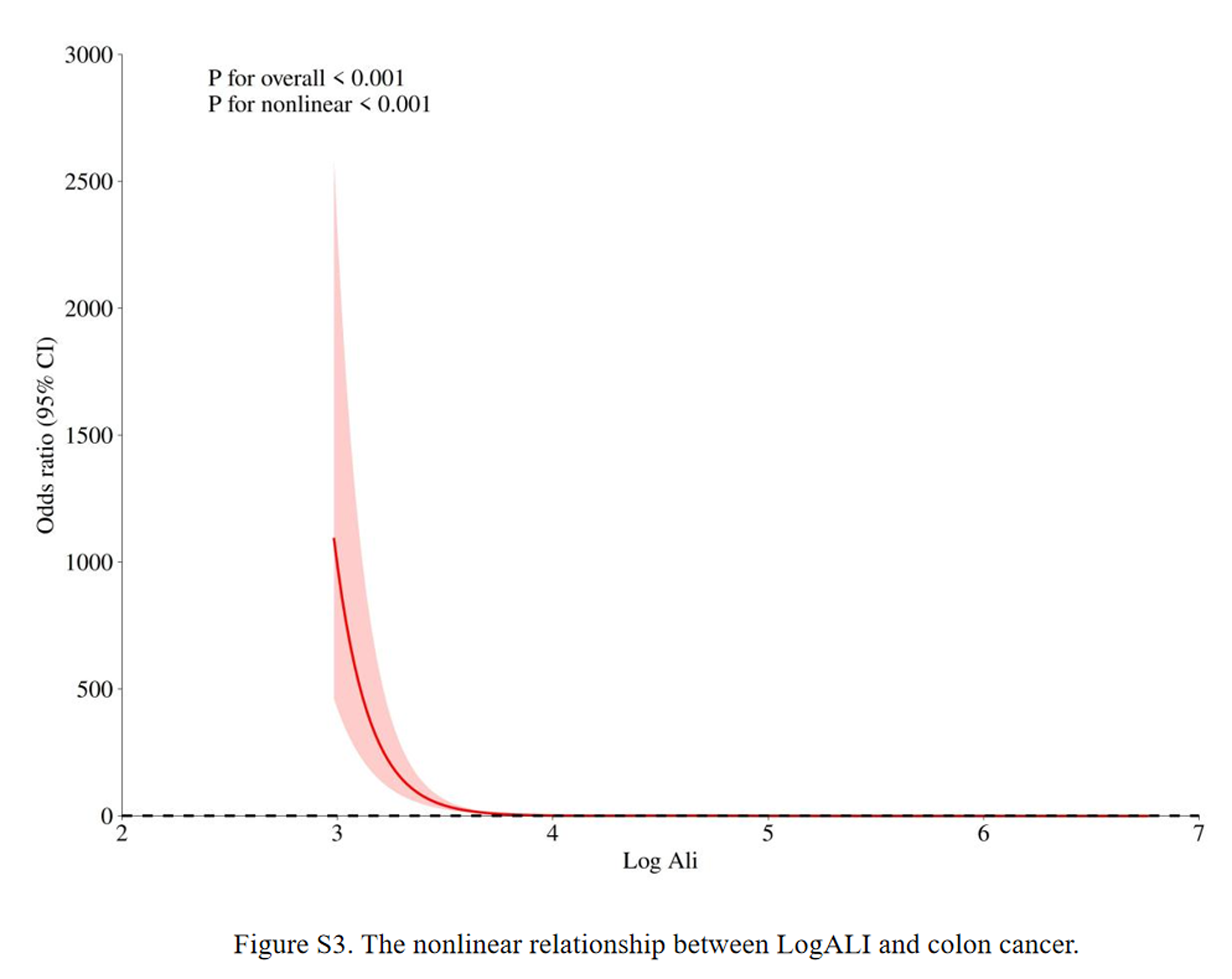

Supplement: Supplementary file 3 [file Image_3.tif]

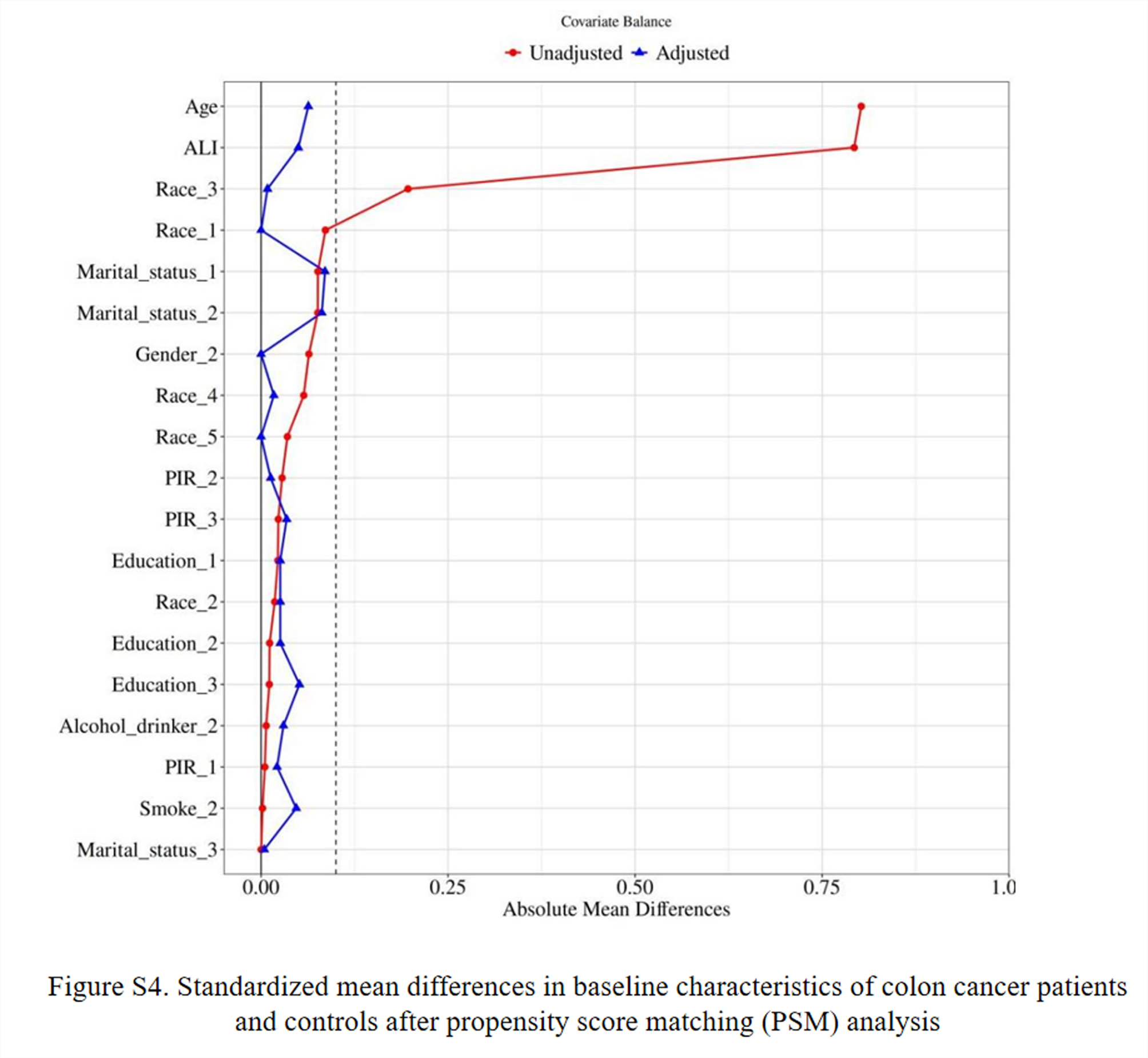

Supplement: Supplementary file 4 [file Image_4.tif]
