# Supplementary material for: Advanced lung cancer inflammation index as a new predictor for colon cancer in elderly patients: an NHANES-based study
Source: Front Nutr. 2025 Sep 4;12:1642913. doi: 10.3389/fnut.2025.1642913 (PMC12445050; doi:10.3389/fnut.2025.1642913)
Supplement: Supplementary file 5 [file Table_1.docx]

Table S1. Threshold effect analysis between LogALI levels and the prevalence of colon cancer.

| Outcome | LogALI | |
| --- | --- | --- |
|  | OR (95%CI)a | P-value |
| Fitting by weighted regression model | 0.04 (0.03 - 0.07) | <.001 |
| Fitting by the weighted two-piecewise regression model |  |  |
| Inflection point | 4.73 |  |
| <4.73 | 0.03 (0.02 - 0.04) | <.001 |
| >4.73 | 0.40 (0.03 - 5.11) | 0.477 |
| p for likelihood ratio test |  | <.001 |

aModel1: Crude.

Note: Missing values for CRP were imputed using mean substitution. For all other variables, records with missing data were excluded from the analysis. log-transformation refers to log base 10 (log₁₀).
