# Supplementary material for: Advanced lung cancer inflammation index as a new predictor for colon cancer in elderly patients: an NHANES-based study
Source: Front Nutr. 2025 Sep 4;12:1642913. doi: 10.3389/fnut.2025.1642913 (PMC12445050; doi:10.3389/fnut.2025.1642913)
Supplement: Supplementary file 6 [file Table_2.docx]

Table S2. Association of advanced logALI among US participants aged 60 to 85 years, NHANES, 1999 to 2020.

| LogALI | Control  (n =9240) | Colon cancer  (n = 248) | Model 1 | P-value | Model 2 | P-value | Model 3 | P-value |
| --- | --- | --- | --- | --- | --- | --- | --- | --- |
| Per ln-unit increase |  |  | 0.03  (0.02 ~ 0.05) | <.001 | 0.05  (0.03 ~ 0.08) | <.001 | 0.05  (0.03 ~ 0.08) | <.001 |
| T1 (<2.95) | 2983 (32.28) | 146  (58.87) | 1.00 (Reference) |  | 1.00 (Reference) |  | 1.00 (Reference) |  |
| T2 (2.95–3.45) | 3165 (34.25) | 62  (25.00) | 0.40  (0.30 ~ 0.54) | <.001 | 0.45  (0.33 ~ 0.62) | <.001 | 0.45  (0.33 ~ 0.61) | <.001 |
| T3 (>3.45) | 3092 (33.46) | 40  (16.13) | 0.26  (0.19 ~ 0.38) | <.001 | 0.33  (0.23 ~ 0.47) | <.001 | 0.33  (0.23 ~ 0.47) | <.001 |
| P for trend |  |  |  | <.001 |  | <.001 |  | <.001 |

Model1: Crude，

Model2: Adjust: Gender, Race, Education, Marital status, PIR, Smoke, Alcohol drinker.

Model3: Adjust: Gender, Age, Race, Education, Marital status, PIR, Smoke, Alcohol drinker.

Note: log-transformation refers to log base 10 (log₁₀).
