# Supplementary material for: Advanced lung cancer inflammation index as a new predictor for colon cancer in elderly patients: an NHANES-based study
Source: Front Nutr. 2025 Sep 4;12:1642913. doi: 10.3389/fnut.2025.1642913 (PMC12445050; doi:10.3389/fnut.2025.1642913)
Supplement: Supplementary file 7 [file Table_3.docx]

Table S3. The relationship between LogALI levels and the prevalence of Colon cancer after PSM

| LogALI | Control  (n = 234) | Colon cancer  (n = 234) | Model 1 | P-value | Model 2 | P-value | Model 3 | P-value |
| --- | --- | --- | --- | --- | --- | --- | --- | --- |
| Per ln-unit increase |  |  | 0.65  (0.41 ~ 1.01) | 0.057 | 0.59  (0.37 ~ 0.95) | 0.031 | 0.61  (0.38 ~ 0.98) | 0.041 |
| T1 (<2.95) | 54  (23.08) | 101  (43.16) | 1.00  (Reference) |  | 1.00 (Reference) |  | 1.00 (Reference) |  |
| T2 (2.95–3.45) | 105 (44.87) | 53  (22.65) | 0.27  (0.17 ~ 0.43) | <.001 | 0.26  (0.16 ~ 0.42) | <.001 | 0.27  (0.17 ~ 0.43) | <.001 |
| T3 (>3.45) | 75  (32.05) | 80  (34.19) | 0.57  (0.36 ~ 0.90) | 0.016 | 0.52  (0.32 ~ 0.85) | 0.009 | 0.53  (0.33 ~ 0.87) | 0.011 |
| P for trend |  |  |  | 0.057 |  | 0.031 |  | 0.041 |

Model1: Crude，

Model2: Adjust: Gender, Race, Education, Marital status, PIR, Smoke, Alcohol drinker.

Model3: Adjust: Gender, Age, Race, Education, Marital status, PIR, Smoke, Alcohol drinker.

Note: log-transformation refers to log base 10 (log₁₀).
