# Supplementary material for: Advanced lung cancer inflammation index as a new predictor for colon cancer in elderly patients: an NHANES-based study
Source: Front Nutr. 2025 Sep 4;12:1642913. doi: 10.3389/fnut.2025.1642913 (PMC12445050; doi:10.3389/fnut.2025.1642913)
Supplement: Supplementary file 8 [file Table_4.docx]

Table S4. The association between ALI and stool type, frequency of bowel movements per week in the overall population and control group.

| Mediating factors | Overall  (n = 12745) | P-value | Controls  (n = 12660) | P-value |
| --- | --- | --- | --- | --- |
|  | β (95% CI)^a^ |  | β (95% CI)^a^ |  |
| stool type | -0.28  (-1.05 ~ 0.49) | 0.475 | -0.27  (-1.04 ~ 0.50) | 0.491 |
| weekly bowel movement frequency | -0.04  (-0.19 ~ 0.10) | 0.572 | -0.05  (-0.19 ~ 0.10) | 0.538 |

Note: Variables include dietary health (DBQ700), stool type (BHQ060), and defecation frequency (BHQ100). “a” indicates adjusted estimates. See Methods section for details.
